# Parameter Estimation of Multiple Frequency-Hopping Signals with Two Sensors

**DOI:** 10.3390/s18041088

**Published:** 2018-04-04

**Authors:** Le Zuo, Jin Pan, Boyuan Ma

**Affiliations:** 1 Department of Microwave Engineering, University of Electronic Science and Technology of China, Chengdu 611731, China; panjin@uestc.edu.cn (J.P.); 201621020208@std.uestc.edu.cn (B.M.); 2 School of Electrical and Electronic Engineering, Nanyang Technological University, 50 Nanyang Avenue, Singapore 639798, Singapore

**Keywords:** DOA estimation, synthetic array, Cramer-Rao lower bound (CRLB), maximum likelihood (ML) estimation, expectation maximization (EM) algorithm

## Abstract

This paper essentially focuses on parameter estimation of multiple wideband emitting sources with time-varying frequencies, such as two-dimensional (2-D) direction of arrival (DOA) and signal sorting, with a low-cost circular synthetic array (CSA) consisting of only two rotating sensors. Our basic idea is to decompose the received data, which is a superimposition of phase measurements from multiple sources into separated groups and separately estimate the DOA associated with each source. Motivated by joint parameter estimation, we propose to adopt the expectation maximization (EM) algorithm in this paper; our method involves two steps, namely, the expectation-step (E-step) and the maximization (M-step). In the E-step, the correspondence of each signal with its emitting source is found. Then, in the M-step, the maximum-likelihood (ML) estimates of the DOA parameters are obtained. These two steps are iteratively and alternatively executed to jointly determine the DOAs and sort multiple signals. Closed-form DOA estimation formulae are developed by ML estimation based on phase data, which also realize an optimal estimation. Directional ambiguity is also addressed by another ML estimation method based on received complex responses. The Cramer-Rao lower bound is derived for understanding the estimation accuracy and performance comparison. The verification of the proposed method is demonstrated with simulations.

## 1. Introduction

Parameter estimation of multiple emitting sources plays an important role in array signal processing due to its applications in various areas, ranging from radar, sonar, microphone arrays, radio astronomy, seismology, medical diagnosis and treatment, to communications [1]. Signals with time-varying frequencies, such as linear frequency-modulation and frequency-hopping (FH) signals, have been extensively used [1,2,3,4,5]. Multiple signals’ sorting and DOA estimations are two important tasks for array signal processing. Lack of prior knowledge of signal sorting and DOA information simultaneously makes multiple FH signals’ parameter estimations challenging.

Radio frequency (RF) DOA estimation is implemented by a direction finding (DF) array, involving multiple sensors placed at different positions in space to receive signals arriving from different directions, and DOAs of these signals are characterized by two parameters, an elevation angle and an azimuth angle. Among the parameter estimation studies, the conventional parametric approaches [6] require that the number of sensors and the associated receivers be more than the number of signals, which leads to an increased hardware complexity in multiple signals’ scenarios. Besides, these methods have a high computation complexity due to the complex eigenvector calculations. Li [7] also proposed an underdetermined narrowband estimator with two sensors, whereas only one-dimensional angular parameter was considered and the sensor separation was half a wavelength to avoid the phase ambiguity. To reduce the number of sensors and receivers, sparse signal representation (SSR) has been recently introduced to signal parameter estimations based on time frequency analysis to exploit the degree of freedom for a sensor array [8,9,10,11,12,13,14,15,16]. Although SSR shows tremendous advantage, it suffers from the grid set, high modeling error, computational efficiency degrade and may lead to unacceptable hardware cost [4]. Furthermore, to achieve high-resolution DOA estimation, a large array aperture must be used, which also limits the utilization of static arrays in the scenarios where the physical size is restricted.

In contrast, a moving array, that is, an array whose element positions vary over time, has been exploited to tradeoff between processing time and hardware cost by time-divided sampling rather than simultaneous sampling as in static arrays. A large number of publications have reported on emitter parameter estimations with moving arrays for improving system performance [17,18,19,20,21,22,23,24,25,26]. Instead of sampling incoming waves by placing many sensors and receiving channels, a passive synthetic array (PSA) comprises an aperture by temporally repositioning a relatively small number of sensors, sweeping a 1-D or 2-D aperture to realize a much larger virtual linear one. For the merits of simple hardware structure and high DF accuracy, linear PSAs have been employed in many applications [18,19,20,21,22]. Although a linear PSA exploits array movement to extend the physical length of a sensor array by using successive measurements in space and time [22], it suffers several shortcomings such as complication in its deployment and control. Alternatively, a circular PSA exploits temporal distribution of baseline diversity to simplify the measurement approach for obtaining necessary information on sources’ DOA [23]. Kawase proposed a method for circular synthetic array (CSA) applications [24], whereas it is only adaptable for a single target and a tracking system, since multiple sources and ambiguity resolution were not considered. Later on, Lan utilized two rotating sensors via Multiple signal classification (MUSIC) algorithms [25]. Nevertheless, only one frequency was considered and the sampling intervals are fixed, which restricts its applications, since in a passive system, sampling intervals and the associated sampling positions are determined by the arriving time of a signal and hence not necessarily uniformly distributed around a circular aperture. Similarly, Liu reported a DOA estimation algorithm via a rotating interferometer [26]. Unfortunately, he ignored the frequency variation of a wideband signal during a rotating period, since the spectrum calculation is under the precondition of single-frequency signals. The frequency variation of a wideband signal must be considered, since the assumption of a single frequency will make the sampling data sparse and the number of sources large. The former can degrade the estimation accuracy and the latter can make signal sorting impractical and incorrect.

In this paper, we herein propose to adopt a low-cost array consisting of only two rotating sensors to tackle the problem of estimating multiple FH signals’ parameters through sample accumulation and the expectation maximization (EM) algorithm. In addition, DOA estimation and phase ambiguity are also considered using the diversities of sensor rotations. As opposed to static arrays, moving arrays accumulate incomplete information about the positions of the emitters successively with tradeoff between processing time and hardware costs. When multiple sources coexist during a rotating period, the signals from these sources are interleaved, only frequency and phase difference can be obtained in each sample, while the signal sorting and DOAs are unknown. For the DOA estimation, signal sorting needs to be known beforehand, since accurate DOA parameters are obtained using the data from the same active source.

An intuitive way to separate multiple sources is the application of the EM algorithm. The main advantage of the EM algorithm is that it provides an iterative solution where a multiple parameter estimation problem is decoupled into several single parameter estimation problem. In particular, we consider a set of received signals from multiple active sources, and the problem of estimating each source’s angular parameters and its emitting source. The use of the EM algorithm for DF was first introduced by Feder and Weinstein [27], who derived a generalized algorithm for the maximum-likelihood (ML) estimation of the DOAs of multiple narrowband signals in noise. Since then, the EM algorithm has been applied to many estimation problems on multiple sources [28,29,30,31,32,33]. Sequence recovery of multiple signals was discussed in [28,29], whereas time and frequency estimations were developed in [30,31]. For the problem of localization of multiple sources, EM-based algorithms were proposed for narrowband signals in [32,33]. Regarding wideband signals, EM algorithms were utilized in [34,35] to estimate multiple sources’ waveforms and localization parameters. The problem studied in this paper is different in the aspect that multiple signals’ distributions are considered in the spatial domain using a moving sensor array. Our method extends the EM algorithm to DOA estimation and sorting for multiple wideband FH signals by a CSA. The idea of our method is based on the decomposition of observed data into different active sources, and estimating the DOA of each source separately. The method iterates back and forth, using the current angular parameters to decompose the observed data hence establishing the correspondence of each signal with its emitting source. Our method involves two steps, namely, the expectation step (E-step) and the maximization step (M-step). In the E-step, the correspondence of each signal with its emitting source are found. Then, in the M-step, the ML estimates of the DOA parameters are obtained.

Furthermore, among the extensive studies of wideband DOA estimation approaches, the ML approach has been regarded as the optimal and robust scheme [34,35]. In this paper, two ML estimators are proposed to calculate the angular parameter of a single source. Since the received signals manifest relative phase differences that contain information about the source’s DOA, phase interferometers have been commonly employed, which utilize measured phase differences across the array to estimate the DOA [36]. The benefit of phase utilization stems from the fact that the phase distribution of a source is a linear projection of wave vector, which contains the angular parameters of a source, onto the position vector determined by sensor positions. This formulation implies that the phases are linear to sampling positions and hence the angular parameters can be extracted from phase measurements in an easy way. Accordingly, in this paper, the DOA estimation is manipulated by a ML estimator based on measured phase differences. Since the phase distribution on a circular aperture is linear to triangular functions of the incident angles, the formulae are in closed form. Accuracy analysis also reveals that the phase-based ML estimator approaches the Cramer Rao lower bound (CRLB).

Additionally, as far as the spatial information is concerned, directional ambiguity is generally encountered, which stems from the fact that the measurement of phase difference can only be made modulo of 2*π* [37]. To solve phase ambiguity, additional sensors are required, thus burdening hardware complexity. This in turn may render the applicability of the systems very unattractive, especially in RF applications where the receiver cost is high. For the sake of ambiguity resolution, low accuracy angle estimation is generally first processed, and then the coarse angle estimation is utilized as a reference for accurate angle estimation [37,38]. Unfortunately, these existing methods [37,38,39] were developed for linear static arrays and cannot be directly applied to a CSA. For a moving array, sensor position diversities may also result in a virtual array that exploits different geometries to resolve the directional ambiguity. Consequently, in this paper, the ambiguity resolution of phase wrapping is also accomplished by another ML estimation based on unambiguous complex responses observed at different positions. Since the complex-response-based ML estimator is nonlinear with the 2-D incident angles, grid search is needed. Hence, the complex-response-based ML estimator is first applied for coarse angle computation, and then the closed-form phase-based ML estimator is employed for refined angle calculation to compromise between computational complexity and estimation accuracy.

The main contributions of this paper can be categorized as follows:

1. Only two sensors and associated receivers are implemented to estimate the parameters of multiple signals with time-varying frequencies by a moving array configuration.
2. Multiple signals’ separation and DOA estimation are realized jointly, by decomposing a complicated multi-parameter optimization into several separated 1-D optimizations.
3. Based on the ML principle, two DOA estimators are developed for ambiguity resolution and closed-form formulae of FH signals.
4. Explicit estimation CRLBs are derived for non-uniform sampling of FH signals both for understanding the accuracy of a CSA and performance comparison.

The rest of this paper is organized as follows. Section 2 establishes the signal model of multiple FH sources. In Section 3, DOA estimation and phase ambiguity resolution are presented for a single wideband source with time-varying frequencies. The CRLB and accuracy analysis are addressed in Section 4. Section 5 develops an EM algorithm for signal separations and DOA estimations of FH signals from multiple emitting sources. Numerical simulations are presented in Section 6. Section 7 concludes this paper.

## 2. Model Formulation

This section addresses the structure of a CSA and establishes the signal model of system responses to the receiving signals from multiple sources with time-varying frequencies.

### 2.1. Circular Synthetic Aperture System Structure

**Figure 1:**
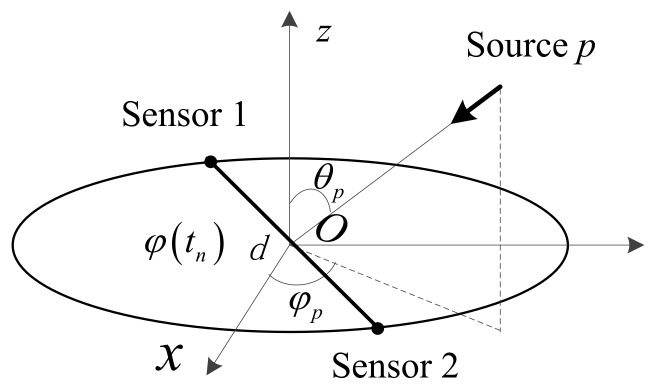
A circular synthetic array (CSA) structure.

The structure of a two-element CSA is briefly depicted in Figure 1. An arm of length *d* with two sensors at each end rotates around its center. Without loss of generality, the rotating center is located at the origin of a Cartesian coordinate and the arm is assumed to rotate within the *xoy*-plane. In a rotating period, the CSA receives *N* measurements of phase differences from *P* far-field sources. Directions of the sources are assumed to be fixed, but frequencies may change within the bandwidth of the signal during a rotating period. When a pulse signal is detected at the rotating orientation $\varphi \left(t_{n}\right)$, with $\varphi \left(t_{n}\right)\in [0,2\pi )$ measured counterclockwise from the *x* axis and $t_{n}$ the sampling interval, the receivers measure the phase difference between the two sensors and the position vector is denoted as $R=d\left(\cos\left(\varphi \left(t_{n}\right)\right){\hat{a}}_{x}+\sin\left(\varphi \left(t_{n}\right)\right){\hat{a}}_{y}\right)$, where ${\hat{a}}_{x}$ and ${\hat{a}}_{y}$ denote unit vectors along *x* and *y* axis, respectively. Since the measurement interval for a pulse signal is very short, the arm orientation variation can be neglected, and each phase measurement is corresponded to a certain orientation. Note that the sampling orientation is determined by the arriving time of a signal, and hence not necessarily uniformly distributed around a circular aperture. Each pulse signal is sampled at its front edge to avoid multiple signal overlapping. It is also assumed in this paper that the receivers’ observation band is wide enough to accommodate the frequency bandwidth of the wideband signals.

### 2.2. Signal Model

The wave vector of the *p*th source at the *n*th sample is represented as $k_{p}=k_{p}\left(n\right)\left(\sin\theta_{p}\cos\varphi_{p}{\hat{a}}_{x}+\sin\theta_{p}\sin\varphi_{p}{\hat{a}}_{y}+\cos\theta_{p}{\hat{a}}_{z}\right)$, with $\varphi_{p}\in [0,2\pi )$ the azimuth angle, $\theta_{p}\in [0,\pi )$ the elevation angle, $k_{p}\left(n\right)=2\pi f_{p}\left(n\right)/c$ the wave number, $f_{p}\left(n\right)$ the instantaneous frequency of the *n*th sample, and *c* the speed of the incoming wave. Then the noiseless and unambiguous phase difference at $\varphi \left(t_{n}\right)$ is observed by the inner product of the wave vector and the position vector, namely

(1) $$\phi_{p}\left(n\right)=k_{p}\cdot R=k_{p}\left(n\right)d\sin\theta_{p}\cos\left(\varphi \left(t_{n}\right)-\varphi_{p}\right)$$

Since a typical implementation of a passive DF system involves a dedicated signal acquisition channel (receiver, analog-to-digital converter, et al.) for each sensor, when the unambiguous phases as indicated in (1) are sampled, phase measurements are contaminated by noises, arising from the presence of receiver noises, hardware imperfections, front end noises, et al. [40]. Hence, the *n*th unambiguous phase sample is expressed as

(2) $$\psi_{p}\left(n\right)=\phi_{p}\left(n\right)+\varepsilon \left(n\right),\;n=1,\ldots ,N$$

where ε(n) represents phase measurement noises. In each phase sample, the ambiguous phase and the wave number can be obtained with reference to the corresponding orientation.

When the sensor separation is greater than half a wavelength, phase ambiguity occurs. Since phase measurements can only be made module of 2π, the sampled ambiguous phase within [−π,π) is given by

(3) $${\tilde{\psi }}_{p}\left(n\right)=\psi_{p}\left(n\right)+2\pi h\left(n\right)$$

where h(n) is an integer and recognized as the ambiguous number.

When *P* sources coexist during a rotating period, the signals from these sources are interleaved and let the observed data be a sum of *P* separate data sets, namely

(4) $$\Phi \left(n\right)=\sum_{p=1}^{P}k_{p}\left(n\right)d\sin\theta_{p}\cos\left(\varphi \left(t_{n}\right)-\varphi_{p}\right)+\varepsilon \left(n\right)+2\pi h\left(n\right)\;$$

The frequencies of the signals and hence $k_{p}\left(n\right)$ in (4) are assumed to be accurately estimated using a number of well-known techniques [41], and this paper assumes that the frequency estimation errors are negligible. Therefore, the angular parameters represented by $\theta_{p}$ and $\varphi_{p}$, along with the frequency sequence, represented by $k_{p}\left(n\right)$, are to be estimated.

## 3. DOA Estimation

As shown in the Section 2, the first term in (2) depends on the rotating orientation and contains information about the unknown DOA parameters, while the second term denotes instrumental noises in phase measurements. Several reasonable assumptions are made about the instrumental phase noises: (a) the phase noises are zero-mean Gaussian noises with variance $\sigma^{2}$, (b) the probability density function (PDF) is the same for all phase noises, (c) the phase noises are mutually independent.

### 3.1. Phase-Based ML Estimation of 2-D DOA

Assuming the phase measurement noises are statistically independent, the conditional PDF for a set of unambiguous phases, i.e., $\Psi_{p}=\left[\Psi_{p}\left(1\right),\;\Psi_{p}\left(2\right),\ldots ,\Psi_{p}\left(N\right)\right]$, for given values of parameters $\theta_{p}=\left(\theta_{p},\varphi_{p}\right)$, can be written as

(5) $$p\left(\Psi_{p}|\theta_{p}\right)=\prod_{n=1}^{N}p\left[\psi_{p}\left(n\right)-k_{p}\left(n\right)d\sin\theta_{p}\cos\left(\varphi \left(t_{n}\right)-\varphi_{p}\right)\right]$$

Taking derivatives of the logarithm of the conditional PDF described by (5) and equate them to zero, the estimate of $\left(\theta_{p},\varphi_{p}\right)$ is then the value $\left({\hat{\theta }}_{p},{\hat{\varphi }}_{p}\right)$ for which the equations

(6) $${\partial \log p\left(\psi_{p}\left(1\right),\psi_{p}\left(2\right),\cdots ,\psi_{p}\left(N\right)|\theta_{p},\varphi_{p}\right)/\partial \theta_{p}|}_{\theta_{p}={\hat{\theta }}_{p}}=0,$$

(7) $${\partial \log p\left(\psi_{p}\left(1\right),\psi_{p}\left(2\right),\cdots ,\psi_{p}\left(N\right)|\theta_{p},\varphi_{p}\right)/\partial \varphi_{p}|}_{\varphi_{p}={\hat{\varphi }}_{p}}=0$$

Equations (6) and (7) hold. From (5) and (6), we have

(8) $$d\sin\theta_{p}\sum_{n=1}^{N}k_{p}{\left(n\right)}^{2}\cos^{2}\left(\varphi \left(t_{n}\right)-\varphi_{p}\right)=\sum_{n=1}^{N}\psi_{p}\left(n\right)k_{p}\left(n\right)\cos\left(\varphi \left(t_{n}\right)-\varphi_{p}\right)$$

Similarly, from (5) and (7), we have

(9) $$d\sin\theta_{p}\sum_{n=1}^{N}k_{p}{\left(n\right)}^{2}\cos\left(\varphi \left(t_{n}\right)-\varphi_{p}\right)\sin\left(\varphi \left(t_{n}\right)-\varphi_{p}\right)=\sum_{n=1}^{N}\psi_{p}\left(n\right)k_{p}\left(n\right)\sin\left(\varphi \left(t_{n}\right)-\varphi_{p}\right)$$

After some manipulations, we obtain the explicit formula for estimates of $\theta_{p},\;\varphi_{p}$, i.e., ${\hat{\theta }}_{p},\;{\hat{\varphi }}_{p}$, namely

(10) $${\hat{\theta }}_{p}=\sin^{-1}\left(\left|b_{1}+jb_{2}\right|/d\right)$$

(11) $${\hat{\varphi }}_{p}=\arg\left(b_{1}+jb_{2}\right)$$

where $b_{1}$ and $b_{2}$ are two elements of the matrix **b**, represented by $b={\left[b_{1},b_{2}\right]}^{T}$, which can be obtained in the matrix form from

(12) $$b={\left(\det\left(A\right)\right)}^{-1}\left[A\right]{\left[c_{1}\;c_{2}\right]}^{T}$$

where det(**A**) denotes the determinant of the matrix **A**, and the matrix elements in $A=\left[{\left(a_{11}a_{12}\right)}^{T}{\left(a_{21}a_{22}\right)}^{T}\right]$ are $a_{11}=\overset{N}{\underset{n=1}{\sum }}k_{p}{\left(n\right)}^{2}\sin^{2}\left(\varphi \left(t_{n}\right)\right)$, $a_{12}=a_{21}=-\overset{N}{\underset{n=1}{\sum }}k_{p}{\left(n\right)}^{2}\sin\left(\varphi \left(t_{n}\right)\right)\cos\left(\varphi \left(t_{n}\right)\right)$, $a_{22}=\overset{N}{\underset{n=1}{\sum }}k_{p}{\left(n\right)}^{2}\cos^{2}\left(\varphi \left(t_{n}\right)\right)$, respectively, and $c_{1}=\overset{N}{\underset{n=1}{\sum }}\psi_{p}\left(n\right)k_{p}\left(n\right)\cos\left(\varphi \left(t_{n}\right)\right)$, $c_{2}=\overset{N}{\underset{n=1}{\sum }}\psi_{p}\left(n\right)k_{p}\left(n\right)\sin\left(\varphi \left(t_{n}\right)\right)$. The phase-based ML estimation for 2-D DOA is in closed form and explicit, yet at the expense of phase ambiguity.

### 3.2. Ambiguity Resolution

Alternatively, complex responses of receiving signals can avoid the phase wrapping problem, since $\exp\left(j\tilde{\psi }\left(n\right)\right)=\exp\left(j\psi \left(n\right)\right)$. The set of complex responses is expressed as

(13) $$y_{p}\left(n\right)=\exp\left(j\psi_{p}\left(n\right)\right)=\exp\left(j\phi_{p}\left(n\right)\right)\exp\left(j\varepsilon \left(n\right)\right)=\exp\left(j\phi_{p}\left(n\right)\right)\left(1+w\left(n\right)\right)$$

where w(n)=exp(jε(n))−1=x(n)+jz(n), $x\left(n\right)=\cos\left(\varepsilon \left(n\right)\right)-1≃-\varepsilon {\left(n\right)}^{2}/2$, z(n)=sin(ε(n))≃ε(n), according to the first-order approximation of Taylor series. Hence, the counterpart of (5) can be rewritten as a conditional PDF for the set of complex responses $y_{p}\left(n\right)=\left[y_{p}\left(1\right),\;y_{p}\left(2\right),\ldots ,y_{p}\left(N\right)\right]$ for given values of parameters $\theta_{c}=\left(\theta_{c},\varphi_{c}\right)$, namely

(14) p(yp|θc)=∏n=1Np(1−Re[yp(n)exp(−jkp(n)dsinθccos(φ(tn)−φc))])

where Re[*x*] denotes the real part of *x*. The ML estimation of $\left(\theta_{c},\varphi_{c}\right)$ is the value $\left({\hat{\theta }}_{c},{\hat{\varphi }}_{c}\right)$ that minimize $p\left(y_{p}|\theta_{c}\right)$ when $y_{p}$ is the complex response vector. Dropping some constant terms, the minimum of $\ln p\left(y_{p}|\theta_{c}\right)$ will occur at the minimum of

(15) L=∑n=1NRe[yp(n)exp(−jkp(n)dsinθccos(φ(tn)−φc))]

Observe that the minimum of L is non-linear with $\theta_{c}$, and hence grid search is required to find the estimated angular parameters that minimize L. The unambiguous estimates of elevation and azimuth angles, that is, ${\hat{\theta }}_{c}=\left({\hat{\theta }}_{c},{\hat{\varphi }}_{c}\right)$, can be used as a coarse angle estimation for the refined and closed-form angle estimation of the phase-based ML estimation, after calculating the ambiguous numbers according to (3), namely

(16) $$\hat{h}\left(n\right)=\mathrm{round}\left[\frac{{\tilde{\psi }}_{p}\left(n\right)-k_{p}\left(n\right)d\sin{\hat{\theta }}_{c}\cos\left(\varphi \left(t_{n}\right)-{\hat{\varphi }}_{c}\right)}{2\pi }\right]$$

where round[*x*] represents the nearest integer of *x*. Then the unambiguous phase can be expressed as

(17) $$\psi_{p}\left(n\right)={\tilde{\psi }}_{p}\left(n\right)-2\pi \hat{h}\left(n\right)$$

Therefore, enhanced DOA estimation accuracy can be obtained based on successful phase ambiguity resolution, adopting (10) and (11).

Regarding the steps in the grid search, excessively dense grids will burden the computational complexity, while too coarse grids can make the ambiguity numbers incorrect. The criteria is analysed as follows. The sufficient condition for correct DOA estimation is that the difference between the phase measurement and the phase distribution determined by the coarse angle estimation does not exceed π, i.e.,

(18) $$\left|{\tilde{\psi }}_{p}\left(n\right)-k_{p}\left(n\right)d\sin{\hat{\theta }}_{c}\cos\left(\varphi \left(t_{n}\right)-{\hat{\varphi }}_{c}\right)\right|<\pi$$

Letting ${\hat{\theta }}_{c}=\theta_{p}+\Delta \theta$ and ${\hat{\varphi }}_{c}=\varphi_{p}+\Delta \varphi$, where Δθ and Δφ are the grid step of the elevation angle and the azimuth angle, respectively and considering (2), we have

(19) $$\frac{-\pi }{k_{p}\left(n\right)d}-\varepsilon \left(n\right)<\sin\theta_{p}\cos\left(\varphi \left(t_{n}\right)-\varphi_{p}\right)-\sin\left(\theta_{p}+\Delta \theta \right)\cos\left(\varphi \left(t_{n}\right)-\varphi_{p}-\Delta \varphi \right)<\frac{\pi }{k_{p}\left(n\right)d}-\varepsilon \left(n\right)$$

Using Taylor expansion of the sine and the cosine functions and neglecting higher orders leads to

(20) $$\frac{-\pi }{k_{p}\left(n\right)d}-\varepsilon \left(n\right)<\cos\theta_{p}\cos\left(\varphi \left(t_{n}\right)-\varphi_{p}\right)\Delta \theta +\sin\theta_{p}\sin\left(\varphi \left(t_{n}\right)-\varphi_{p}\right)\Delta \varphi <\frac{\pi }{k_{p}\left(n\right)d}-\varepsilon \left(n\right)$$

It can be seen from (20) that the grid step is dependent on the electrical size of the array, phase noise and the incident elevation angle. Moreover, for a small elevation angle, the elevation grid step should be small, while the azimuth grid step could be large.

## 4. Accuracy Analysis

In a measurement system, it is important to derive the best estimation that can be made with available observations. This section derives the CRLB in the absence of phase ambiguity, in order to demonstrate the potential predominance of a CSA in estimation accuracy for 2-D DOA of wideband and time-varying signals. Moreover, accuracy analysis of the phase-based estimator reveals that the proposed method approaches the CRLBs and hence it is an optimal estimation.

### 4.1. DOA Estimation CRLB

According to (1), the Jacobi matrix is given by

(21) $$J=\left[\begin{array}{cc} \begin{array}{l} \frac{\partial \psi_{p}\left(1\right)}{\partial \theta_{p}}\\ \;\vdots \end{array} & \begin{array}{l} \frac{\partial \psi_{p}\left(1\right)}{\partial \varphi_{p}}\\ \;\vdots \end{array}\\ \frac{\partial \psi_{p}\left(N\right)}{\partial \theta_{p}} & \frac{\partial \psi_{p}\left(N\right)}{\partial \varphi_{p}} \end{array}\right]=d\left[\begin{array}{cc} k_{p}\left(1\right)\cos\theta_{p}\cos\left(\varphi \left(t_{1}\right)-\varphi_{p}\right) & -k_{p}\left(1\right)\sin\theta \sin\left(\varphi \left(t_{1}\right)-\varphi_{p}\right)\\ \begin{array}{c} \vdots \\ k_{p}\left(N\right)\cos\theta_{p}\cos\left(\varphi \left(t_{N}\right)-\varphi_{p}\right) \end{array} & \begin{array}{c} \vdots \\ -k_{p}\left(N\right)\sin\theta \sin\left(\varphi \left(t_{N}\right)-\varphi_{p}\right) \end{array} \end{array}\right]$$

Hence, the Fisher information matrix (FIM) is given by [42]

(22) $$F=\frac{1}{\sigma^{2}}J^{T}J=\left[\begin{array}{cc} f_{11} & f_{12}\\ f_{21} & f_{22} \end{array}\right]$$

where $f_{11}={\left(d\cos\theta_{p}/\sigma \right)}^{2}\overset{N}{\underset{n=1}{\sum }}k_{p}{\left(n\right)}^{2}cp_{n}^{2}$, $f_{12}=f_{21}={\left(d/\sigma \right)}^{2}\sin\theta_{p}\cos\theta_{p}\overset{N}{\underset{n=1}{\sum }}k_{p}{\left(n\right)}^{2}cp_{n}\cdot sp_{n}$, $f_{22}={\left(d\sin\theta_{p}/\sigma \right)}^{2}\overset{N}{\underset{n=1}{\sum }}k_{p}{\left(n\right)}^{2}sp_{n}^{2}$, and $cp_{n}=\cos\left(\varphi \left(t_{n}\right)-\varphi_{p}\right)$, $sp_{n}=\sin\left(\varphi \left(t_{n}\right)-\varphi_{p}\right)$. Hence, the CRLBs of the elevation angle and the azimuth angle are evaluated by the inverse of FIM, and given by closed-form expressions for angular parameter estimations of wideband time-varying signals, namely

(23) $$\mathrm{var}\left(\Delta \theta_{p}\right)\geq {\left(\sigma /\left(d\cos\theta_{p}\right)\right)}^{2}\sum_{n=1}^{N}k_{p}{\left(n\right)}^{2}sp_{n}^{2}/\left(\sum_{n=1}^{N}k_{p}{\left(n\right)}^{2}cp_{n}^{2}\sum_{n=1}^{N}k_{p}{\left(n\right)}^{2}sp_{n}^{2}-{\left(\sum_{n=1}^{N}k_{p}{\left(n\right)}^{2}cp_{n}\cdot sp_{n}\right)}^{2}\right)$$

(24) $$\mathrm{var}\left(\Delta \varphi_{p}\right)\geq {\left(\sigma /\left(d\sin\theta_{p}\right)\right)}^{2}\sum_{n=1}^{N}k_{p}{\left(n\right)}^{2}cp_{n}^{2}/\left(\sum_{n=1}^{N}k_{p}{\left(n\right)}^{2}cp_{n}^{2}\sum_{n=1}^{N}k_{p}{\left(n\right)}^{2}sp_{n}^{2}-{\left(\sum_{n=1}^{N}k_{p}{\left(n\right)}^{2}cp_{n}\cdot sp_{n}\right)}^{2}\right)$$

If $\varphi \left(t_{n}\right)$ is approximately uniformly distributed around a circular aperture, then $\overset{N}{\underset{n=1}{\sum }}\cos^{2}\left(\varphi \left(t_{n}\right)-\varphi_{p}\right)≅N/2$, $\overset{N}{\underset{n=1}{\sum }}\sin^{2}\left(\varphi \left(t_{n}\right)-\varphi_{p}\right)≅N/2$, $\overset{N}{\underset{n=1}{\sum }}\sin\left(\varphi \left(t_{n}\right)-\varphi_{p}\right)\cos\left(\varphi \left(t_{n}\right)-\varphi_{p}\right)≅0$, and if the signals approximate narrow-band, the CRLBs of elevation angle and azimuth angle obtained are degenerated to those in [26], namely

(25) $$\mathrm{var}\left(\Delta \theta_{p}\right)\geq 2\sigma^{2}{\left(k_{0}d\cos\theta_{p}\right)}^{-2}/N$$

(26) $$\mathrm{var}\left(\Delta \varphi_{p}\right)\geq 2\sigma^{2}{\left(k_{0}d\sin\theta_{p}\right)}^{-2}/N$$

where $k_{0}$ is the wave number of the narrow-band signal, var(*x*) denotes the variance of *x*. Equations indicate that the lower bounds of DOA estimation are inversely proportional to the number of samples, and as the elevation angle increases, the accuracy of azimuth angle estimation increases, while the accuracy of elevation angle estimation decreases. However, the CRLBs represented by (23) and (24) are more general than (25) and (26), as the former equations take the frequency variation of each signal into account. Hence, they are applicable to wideband and time-varying signals. Moreover, comparing the accuracies of static circular arrays, when the sample number of a CSA is the same as the element number of a static circular array, the two angle estimation accuracies are the same [43].

### 4.2. Accuracy Analysis of the Phase-Based ML Estimator

In order to compare with the CRLBs, estimation accuracy of the phase-based ML estimator is investigated. The noises on **b** are introduced by phase measurement noises, i.e.,

(27) $$\Delta b=\hat{b}-b=\frac{1}{\det\left(A\right)}\left[A\right]{\left[\Delta c_{1}\;\Delta c_{2}\right]}^{T}$$

where $\Delta c_{1}=\overset{N}{\underset{n=1}{\sum }}\varepsilon \left(n\right)k_{p}\left(n\right)\cos\left(\varphi \left(t_{n}\right)\right)$, $\Delta c_{2}=\overset{N}{\underset{n=1}{\sum }}\varepsilon \left(n\right)k_{p}\left(n\right)\sin\left(\varphi \left(t_{n}\right)\right)$. Let $t=b_{1}+jb_{2}$, and on one hand,

(28) $$\Delta t=\Delta b_{1}+j\Delta b_{2}$$

On the other hand, since $t=d\sin\theta_{p}\exp\left(j\varphi_{p}\right)$, first-order approximation of the derivation of *t* yields

(29) $$\Delta t=d\left(\cos\theta_{p}\Delta \theta_{p}+j\sin\varphi_{p}\Delta \varphi_{p}\right)e^{j\varphi_{p}}$$

Comparison of real and imaginary parts of (28) and (29) yields

(30) $$\Delta \theta_{p}=\frac{\cos\varphi_{p}\Delta b_{1}+\sin\varphi_{p}\Delta b_{2}}{d\cos\theta_{p}}$$

(31) $$\Delta \varphi_{p}=\frac{\cos\varphi_{p}\Delta b_{2}-\sin\varphi_{p}\Delta b_{1}}{d\sin\theta_{p}}$$

Considering (27), we get the variances of $\theta_{p}$ and $\varphi_{p}$, respectively,

(32) $$\mathrm{var}\left(\Delta \theta_{p}\right)=\frac{{\left(\sigma /\left(d\cos\theta_{p}\right)\right)}^{2}\left(\cos^{2}\varphi_{p}\sum_{n=1}^{N}k_{p}{\left(n\right)}^{2}\sin^{2}\left(\varphi \left(t_{n}\right)\right)+\sin^{2}\varphi_{p}\sum_{n=1}^{N}k_{p}{\left(n\right)}^{2}\cos^{2}\left(\varphi \left(t_{n}\right)\right)\right)}{\left(\sum_{n=1}^{N}k_{p}{\left(n\right)}^{2}\sin^{2}\left(\varphi \left(t_{n}\right)\right)\sum_{n=1}^{N}k_{p}{\left(n\right)}^{2}\cos^{2}\left(\varphi \left(t_{n}\right)\right)+\sum_{n=1}^{N}k_{p}{\left(n\right)}^{2}\sin\left(\varphi \left(t_{n}\right)\right)\cos\left(\varphi \left(t_{n}\right)\right)\right)}$$

(33) $$\mathrm{var}\left(\Delta \varphi_{p}\right)=\frac{{\left(\sigma /\left(d\sin\theta_{p}\right)\right)}^{2}\left(\cos^{2}\varphi_{p}\sum_{n=1}^{N}k_{p}{\left(n\right)}^{2}\cos^{2}\left(\varphi \left(t_{n}\right)\right)+\sin^{2}\varphi_{p}\sum_{n=1}^{N}k_{p}{\left(n\right)}^{2}\sin^{2}\left(\varphi \left(t_{n}\right)\right)\right)}{\left(\sum_{n=1}^{N}k_{p}{\left(n\right)}^{2}\sin^{2}\left(\varphi \left(t_{n}\right)\right)\sum_{n=1}^{N}k_{p}{\left(n\right)}^{2}\cos^{2}\left(\varphi \left(t_{n}\right)\right)+\sum_{n=1}^{N}k_{p}{\left(n\right)}^{2}\sin\left(\varphi \left(t_{n}\right)\right)\cos\left(\varphi \left(t_{n}\right)\right)\right)}$$

If $\varphi \left(t_{n}\right)$ is approximately uniformly distributed around a circular aperture, and the bandwidth of the signals is zero, the right side of (32) approaches the right side of (25), while the right side of (33) approach the right side of (26), revealing that estimation accuracies of the proposed algorithm approaches the CRLBs and hence the optimal estimation is achieved.

## 5. EM Algorithm

In the multiple sources’ case, the direct ML approach requires the solution to

(34) $$\underset{\begin{array}{l} \theta_{1},\cdots ,\theta_{P}\\ \varphi_{1},\cdots ,\varphi_{P} \end{array}}{\min}\left\{\sum_{n=1}^{N}\mathrm{dis}{\left[\Phi \left(t_{n}\right)-\phi_{p}\left(n\right)\right]}^{2}\right\}$$

where dis[x]=|mod(x,2π)| is the distance defined by the wrap-around phase difference, and mod(x,2π) is the modulo 2π operator of *x*. Note that the correspondence between each receiving signal and the active source that emits this signal is unknown beforehand, as well as the source’s DOA. Therefore, the direct solution to the optimization is a *NP*-hard problem [44].

Alternatively, if the interleaved data could be replaced by independent data groups, then the problem would be decoupled to *P* parallel optimization problems. Assuming the correspondences between the signals and the sources are already known, the estimations of multiple DOA are *P* separated estimation problems that can be solved by the closed-form estimator proposed in Section 3. On the other side, if one source’s DOA is known beforehand, the correspondence can be established by finding the nearest expected noiseless phase difference emitted by the source. Consequently, the idea of our method is based on decomposition of the observed phase data into different groups, and estimate the DOA of each source separately. The procedure of our algorithm consists of an expectation step (E-step) and a maximization step (M-step).

### 5.1. Expectation Step

In each iteration step, the expected phase data are defined by the newly estimated angular parameters as

(35) $$\phi_{p}\left(n;\theta_{p}^{\left(i\right)},\varphi_{p}^{\left(i\right)}\right)=k_{p}\left(n\right)d\sin\theta_{p}^{\left(i\right)}\cos\left(\varphi \left(t_{n}\right)-\varphi_{p}^{\left(i\right)}\right)$$

The E-step is processed by an exclusively binary classification, that is, each phase difference is decomposed into one group, where the distance between the phase difference and the expected phase data is minimized, i.e.,

(36) $${\tilde{\psi }}_{p}^{\left(i\right)}\left(n\right)=\underset{p}{\min}\left\{\mathrm{dis}\left[\Phi \left(n\right)-\phi_{p}\left(n;{\hat{\theta }}_{p}^{\left(i\right)},{\hat{\varphi }}_{p}^{\left(i\right)}\right)\right]\right\}$$

Then the phase data are decoupled into *P* groups.

### 5.2. Maximization Step

After decoupling the phase data as described in the E-step, the phase differences in each group can be applied to the estimation method proposed in Section 3. The updated DOA estimation is obtained from the phase data decomposed by (36), namely

(37) $$\left({\hat{\theta }}_{p}^{\left(i+1\right)},{\hat{\varphi }}_{p}^{\left(i+1\right)}\right)=\underset{\left(\theta_{p},\varphi_{p}\right)}{\max}\log p\left({\tilde{\psi }}_{p}^{\left(i\right)}\left(1\right),{\tilde{\psi }}_{p}^{\left(i\right)}\left(2\right),\cdots ,{\tilde{\psi }}_{p}^{\left(i\right)}\left(N\right)|\theta_{p},\varphi_{p}\right)$$

Then the calculated parameters are used to decompose the data once again in the E-step.

### 5.3. Full Algorithm Summary

**Figure 2:**
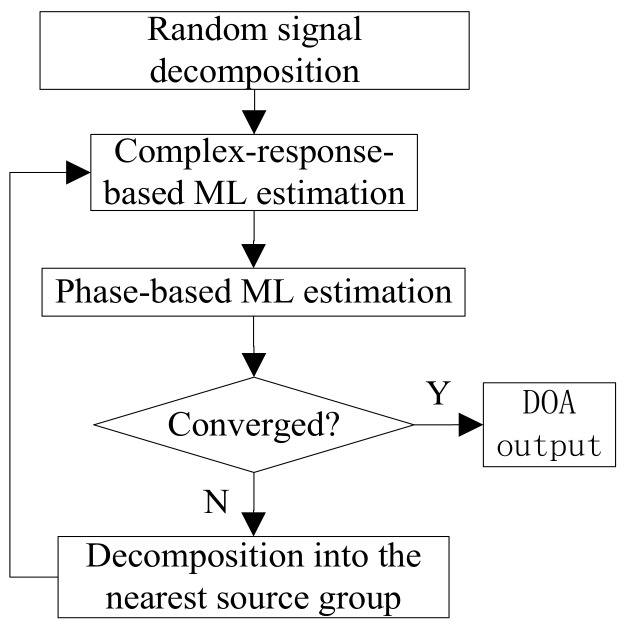
Procedure of direction of arrival (DOA) estimation of multiple wideband sources incorporated with the expectation maximization (EM) algorithm.

The procedure of multiple source DOA estimation incorporated with the EM algorithm is illustrated in Figure 2. The algorithm begins with assigning each signal to one random group that emits. Then, in the iterative EM algorithm, the original *P*-dimensional optimization is reduced to *P* separate one-dimensional optimizations, which is much easier. The algorithm iterates back and forth, using the current parameter estimates to decompose the observed data better, thus improving the next parameter estimates. The algorithm converges to a stationary point of the likelihood function where each iteration cycle increases the likelihood of the estimated parameters.

## 6. Simulation Results

This section carries out simulations to demonstrate the performance of CSAs. The center frequency was fixed at 1 GHz with a bandwidth of 10%, that is, the frequency of each signal was randomly set from 0.95 GHz to 1.05 GHz and the sensor separation was set to be 2 m.

### 6.1. Single-Source Case

**Figure 3:**
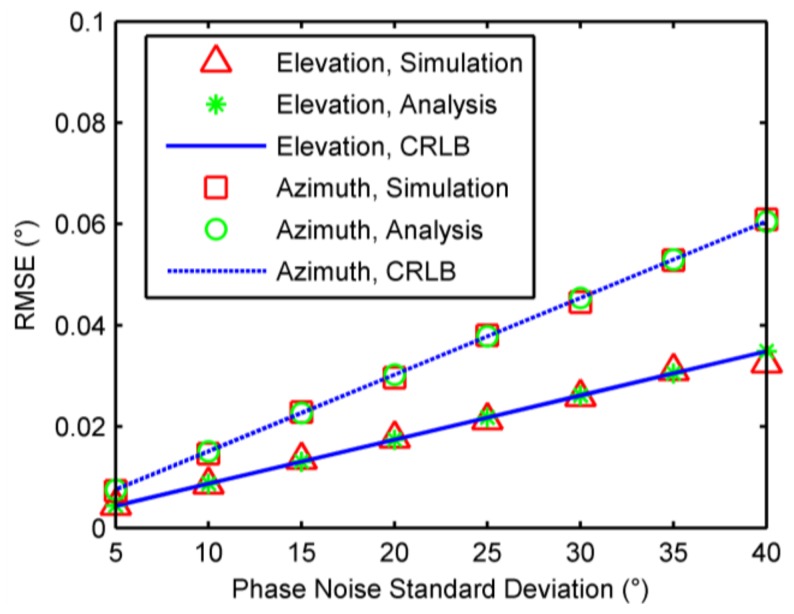
Root mean square errors (RMSEs) against phase noises.

**Figure 4:**
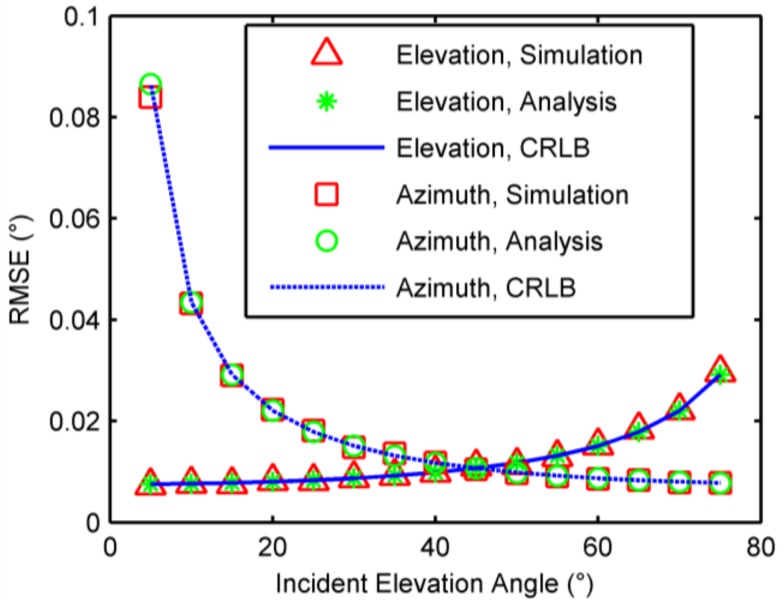
RMSEs against incident elevation angles.

**Figure 5:**
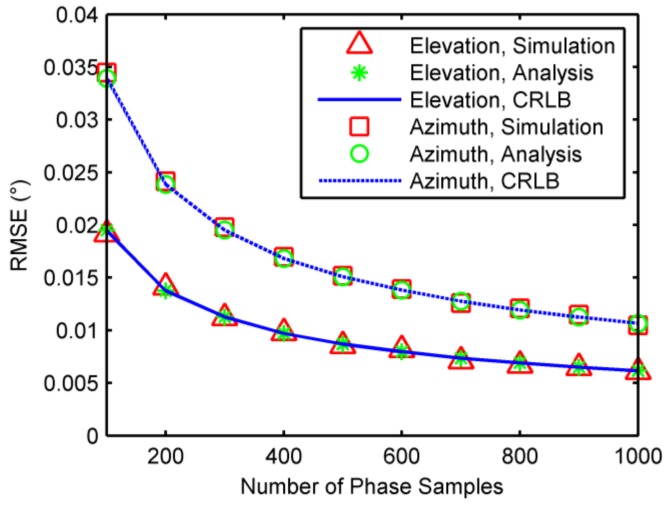
RMSEs against number of phase samples.

When a single source was present, root mean square errors (RMSEs) of 2-D DOA were calculated by 1000 independent trials against phase noises, incident elevation angles, and number of samples, respectively. The CRLBs and analytical results of accuracies as analyzed in Section 4 were also computed for comparison. In the first example, the elevation and azimuth angles were fixed at 30 degrees and 170 degrees, respectively, and the standard deviation of phase noise was from 5 degrees to 40 degrees, along with the number of samples being 500. The RMSEs against phase noises are shown in Figure 3. In the second example, the azimuth angle was fixed at 170 degrees, the number of phase samples was 500, and the standard deviation of phase noise was 10 degrees, while the elevation angle varies from 5 degrees to 75 degrees. The RMSEs against incident elevation angles are illustrated in Figure 4. In the third example, the elevation and azimuth angles were fixed at 30 degrees and 170 degree, respectively, and the standard deviation of phase noise was 10 degrees. The number of samples were chosen from 100 to 1000. The RMSEs against the number of phase samples are plotted in Figure 5. As seen Figure 3, Figure 4 and Figure 5, accuracy increases as decrease of the elevation angle, and as increase of the azimuth angle and the number of samples. Meanwhile, the results agree with the analysis in Section 4 and approach the CRLBs.

### 6.2. Multiple-Source Case

**Figure 6:**
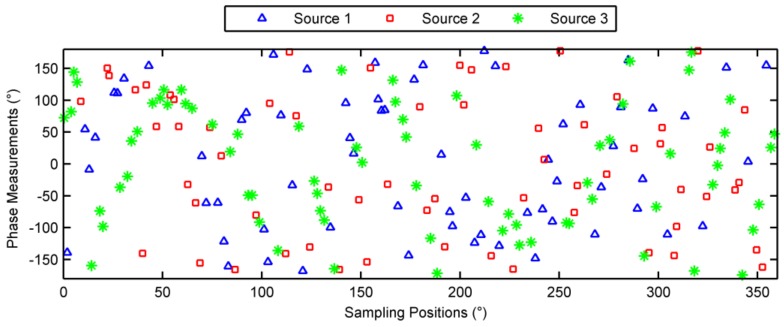
Sampled phases from three sources.

**Figure 7:**
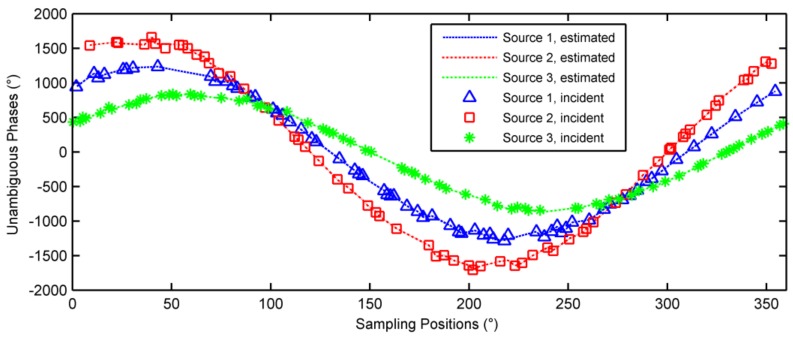
Unambiguous phases of the decomposed signals.

Consider three sources located at $\left(\theta_{1},\varphi_{1}\right)=\left(15{}^{\circ},40{}^{\circ}\right)$, $\left(\theta_{2},\varphi_{2}\right)=\left(20{}^{\circ},30{}^{\circ}\right)$ and $\left(\theta_{3},\varphi_{3}\right)=\left(10{}^{\circ},60{}^{\circ}\right)$, respectively. 200 pulses were randomly sampled during a rotation period. The phases noises were Gaussian noises with zero mean and 5 degrees variance. The observed phases are depicted in Figure 6 with different symbols denoting these three sources, and also note that the associations between each sample and its source are not known yet. The estimation results were converged after 4 iterations, the unambiguous phases of the incident signals and the decomposed signals are shown in Figure 7. As seen in Figure 7, the three sources were separated successfully. The estimated incident angles were $\left({\hat{\theta }}_{1},{\hat{\varphi }}_{1}\right)=\left(15.0004{}^{\circ},\;39.9751{}^{\circ}\right)$, $\left({\hat{\theta }}_{2},{\hat{\varphi }}_{2}\right)=\left(20.0004{}^{\circ},\;29.997{}^{\circ}\right)$, and $\left({\hat{\theta }}_{3},{\hat{\varphi }}_{3}\right)=\left(10.0017{}^{\circ},60.0558{}^{\circ}\right)$, respectively. The DOA estimation has very high accuracy.

**Figure 8:**
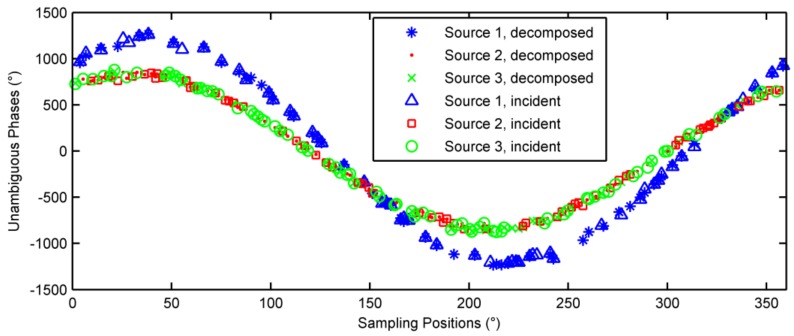
Unambiguous phases of closely located sources.

**Figure 9:**
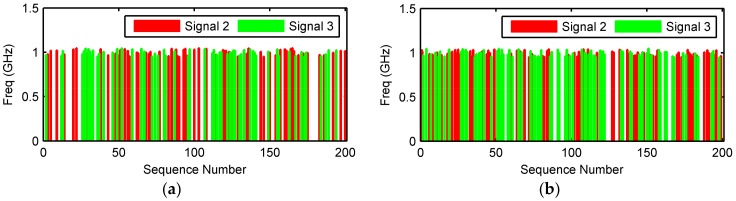
Signal decomposition of two closely located sources. (**a**) Incident, (**b**) Decomposed.

In the second example, two of the three sources are located closely, e.g., $\left(\theta_{1},\varphi_{1}\right)=\left(30{}^{\circ},40{}^{\circ}\right)$, $\left(\theta_{2},\varphi_{2}\right)=\left(20{}^{\circ},30{}^{\circ}\right)$ and $\left(\theta_{3},\varphi_{3}\right)=\left(20.5{}^{\circ},30{}^{\circ}\right)$. The number of pulses remain 200 during a rotation period. The phases noises were Gaussian noises with zero mean and 20 degrees variance. 19 iterations were run to obtain convergence and the unambiguous phases of the incidents signals and the decomposed signals are shown in Figure 8. The estimated incident angles were $\left({\hat{\theta }}_{1},{\hat{\varphi }}_{1}\right)=\left(30.2662{}^{\circ},\;39.9335{}^{\circ}\right)$, $\left({\hat{\theta }}_{2},{\hat{\varphi }}_{2}\right)=\left(20.0002{}^{\circ},\;30.4008{}^{\circ}\right)$ and $\left({\hat{\theta }}_{3},{\hat{\varphi }}_{3}\right)=\left(20.7954{}^{\circ},29.0044{}^{\circ}\right)$, respectively. The signal decomposition of two closely located sources, source 2 and source 3, is shown in Figure 9. As seen from Figure 9, the correspondence of signals and sources are incorrect for source 2 and source 3. The reason is that when the phase differences between two closely separated sources are smaller than the phase noise, the two sources are merged into one.

## 7. Conclusions

In this paper, it has been shown that a CSA consisting of only two sensors is suitable for application of parameter estimations in complex signal scenarios including time-varying signals’ sorting and multiple active sources’ DOA estimations. The EM algorithm has been utilized in the sense of multiple sources’ joint decomposition and parameter estimation. The most attractive feature of the proposed EM algorithm is that the full dimensional optimization has been decouples into several parallel low-dimensional parameter subspaces and multiple sources’ DOAs are estimated separately. Hence, it is very efficient computationally. A phase-based ML estimator for wideband FH signals has been addressed in closed form and also approaches the optimal estimation. Meanwhile, a complex-response-based ML estimator has been developed to resolve phase ambiguity. Simulation results have shown the effectiveness and appealing performance of the proposed algorithm.
